# Dietary iron intakes and odds of iron deficiency anaemia among pregnant women in Ifako-Ijaiye, Lagos, Nigeria: a cross-sectional study

**DOI:** 10.11604/pamj.2022.42.23.29965

**Published:** 2022-05-11

**Authors:** Temitope Elizabeth Adeboye, Ifeoluwa Omolara Bodunde, Akinkunmi Paul Okekunle

**Affiliations:** 1 Department of Nutrition and Dietetics, University of Ibadan, Ibadan, Oyo State, Nigeria,; 2 The Postgraduate College University of Ibadan, University of Ibadan, Ibadan, Nigeria,; 3 Nutritional Epidemiology Laboratory, College of Human Ecology, Seoul National University, Seoul, Korea

**Keywords:** Iron, haemoglobin, anaemia, pregnancy, Nigeria

## Abstract

**Introduction:**

iron deficiency anaemia (IDA) in pregnancy is a prominent risk factor for maternal mortality and poor pregnancy outcomes in low- and middle-income countries, but studies on the significance of dietary iron intake (DII) in IDA in this population are limited. This study assessed the association of DII with odds of IDA among pregnant women attending primary health centres in Ifako-Ijaiye, Lagos, Nigeria.

**Methods:**

sociodemographic information and dietary intakes were assessed among 432 singleton pregnant women (without pre-existing medical complications) using a pre-tested questionnaire and 24 hr-dietary recall, respectively. Dietary iron intake (DII) was derived from the 24 hr-dietary recall using the West African food composition table. Haemoglobin (Hb) levels in blood samples were measured using the haemoglobin-cyanide technique, and IDA was defined using the World Health Organization criteria. Multivariable-adjusted odds ratios (OR) of IDA and 95% confidence intervals (CI) by energy-adjusted tertiles of DII were estimated using logistic regression with a two-sided P<0.05.

**Results:**

mean age of respondents was 28.5 ± 4.6 years, and the average gestational age was 31.3 ± 4.1 weeks. Mean DII was 20.3 ± 3.3 mg/day, and Hb concentration was 97.9 ± 12.9 g/L. Furthermore, 83.8% had IDA and multivariable-adjusted OR and 95% CI for odds of IDA across tertiles of energy-adjusted DII were 1.00, 0.32 (0.05, 1.77), 0.07 (0.01, 0.36) P for trend < 0.0001 adjusting for age, primigravidae status and monthly income.

**Conclusion:**

higher DII was inversely associated with the odds of IDA among pregnant women. Behavioural change communication promoting the consumption of iron-rich foods might be a viable dietary strategy to alleviate the high burden of IDA among women in this population.

## Introduction

Anaemia represents a health condition where blood haemoglobin (Hb) concentration is lower than normal [1]. It is a public health problem that affects about a third of the global population [2] and is associated with several complications [3]. Anaemia exists in different forms, but iron deficiency anaemia (IDA) appears to be the most common form of anaemia [2] and is associated with high rates of complications in pregnancy [3] and maternal mortality [4,5]. It accounts for almost 115,000 maternal deaths and over three million disability-adjusted life years worldwide [6].

Specific reports in Africa have explored the burden of IDA [7-12], with minimal information on the level of dietary iron intake (DII). This information is necessary to discern the contributions of DII to the odds of IDA among pregnant women. Also, information on the factor(s) related to DII and IDA among pregnant women is relatively scarce. Discerning sociodemographic and lifestyle factors associated with poor DII and depleted maternal iron stores cannot be over-estimated in improving pregnancy and birth outcomes. Understanding the factor(s) associated with DII and IDA among pregnant women is vital to advance local understanding of maternal health and design context-specific intervention(s) to address the burden of IDA among pregnant women. Also, exploring the relationship between DII and IDA might be of interest in providing evidence to support health promotion advice in improving maternal health. This information might help improve pregnancy outcomes and manage the burden of IDA-related morbidity and mortality.

Therefore, we hypothesized a null relationship between DII and the odds of IDA among pregnant women. Also, we postulated that sociodemographic factors are not associated with DII and IDA in the same population. This study assessed the association of DII with IDA among singleton pregnant women in Ifako-Ijaiye, Lagos, Nigeria.

## Methods

**Ethics statement:** the University of Ibadan/University College Hospital Nigeria Ethics Committee (UI/EC/09/0008) approved the study and written informed consent was obtained from all respondents before taking part in the study.

**Study design, sample size determination, sampling and respondent enrollment:** healthy singleton pregnant women were enrolled from the eight primary health centres (PHC) in Ifako-Ijaiye local government area (LGA) of Lagos State, Nigeria, through multi-stage random sampling. Ifako-Ijaiye is situated on latitude 6°52´ 0” N and longitude 2°53´ 60” E covering 43 km^2^ of the 3,577 km^2^ land area of Lagos State, Nigeria. It comprises sixteen settlements with an estimated population of 427,878 [13].

Using the Kish equation for sample size estimation for cross-sectional studies [14], we estimated a minimum sample size of n=530 in this study. We applied a 55.8% prevalence of IDA among pregnant women in Africa [15,16], a two-sided 95% confidence level, an absolute error of 5% and a type I error of 5%. Also, we forecasted an additional 40% non-response rate. Permission to conduct the study was obtained from PHC. The sample size was proportionally allocated across the eight PHCs in Ifako Ijaye LGA. We adopted a systematic sampling to administer the number of respondents recruited from a sampling frame of pregnant women presenting for regular antenatal visits at each PHC [17]. Respondents were eligible for inclusion if they met the following criteria: 1) singleton pregnancy; 2) with gestational age (GA) ≥ 25 weeks; 3) without pre-existing medical complication(s) and; 4) regularly attending ante-natal clinic at the PHC. Pregnant women were invited to participate during antenatal visits to the PHC, educated about the study and enrolled after obtaining written informed consent. Information on sociodemographic characteristics and diet (using a 24-hour dietary recall) were collected by trained personnel using an interviewer-administered questionnaire in English or Yoruba languages.

**Sociodemographic characteristics:** respondents provided information on sociodemographic characteristics through in-person interviews conducted by trained personnel. The age of respondents at last birthday was reported in years and categorized as '16 - 24 years' or '25 years'. Respondent reported the highest formal education completed and was classified ‘at least secondary education´ or 'tertiary education', marital status was presented as single, married, widow or separated and reported as 'never married', or 'married' and average monthly income was reported in naira (N) and classified as '≤ N16,000' or > 'N16,000'. Gestational age (in weeks) was self-reported but validated using the last menstrual period reported in the medical record. Also, respondents provided information on whether the current pregnancy was the first ('no' or 'yes') and the number of birth experiences (parity) and classified as '≤ 2' or '>2'.

**Dietary iron intake and assessment - predictor:** dietary intake of foods of respondents was assessed using a 24-hour dietary recall questionnaire [18-20] administered by trained personnel to secure information on the foods and drinks consumed in the last 24 hours before recruitment. The amount of food (grams) and drinks (ml) consumed per unit of time was provided, and nutrient intakes, including total energy (kcal/day) and iron intakes (mg/day) of food items consumed, were estimated by multiplying the daily consumption of each food with the nutrient content of a specified amount using the West African food composition table [21]. Dietary iron intake (DII) data were adjusted for total energy intake using the residual method [22].

**Determination of packed cell volume (PCV):** PCV represents the proportion of the whole blood made up of red cells. Trained phlebotomists took blood samples through a finger prick into a heparinized capillary tube, centrifuged at 3000 rpm for 5 mins using micro haematocrit centrifuge, and PCV was determined using a Hewkley microhematocrit reader [23].

**Determination of haemoglobin (Hb) concentration:** Hb concentration was determined using the haemoglobin-cyanide (HICN) technique reported elsewhere [23]. Briefly, 20μl blood samples drawn via capillary tube were diluted in modified Drabkin´s solution (containing potassium ferricyanide and potassium cyanide) to haemolyze red cells. Hb was trapped through its reaction with ferri-cyanide to form methaemoglobin and converted to HICN. The HICN absorbance was read at a wavelength of 540nm (compared to a reference HICN standard solution) using a spectrophotometer. Hb values were obtained from a direct read-out digital Hb meter.

**Ascertainment of iron deficiency anaemia (IDA) - outcome:** according to the World Health Organization criteria, IDA was defined as blood Hb concentrations less than 110g/L [1]. Respondents with blood Hb concentrations less than 110g/L were classified as IDA cases, while those with blood Hb concentrations of 110g/L or more were classified as non-cases of IDA.

**Statistical analysis:** out of 550 respondents invited for the study, 62 respondents declined participation, medical records of 22 respondents could be accessed, 16 respondents declined to provide blood samples, and 18 respondents with missing data on dietary iron intake were excluded. In all, 432 singleton pregnant women were included in the final analysis of this sample. Categorical and continuous variables were presented using frequency (percentages) and mean ± SD, respectively. DII were adjusted for energy intakes using the residual method and categorized by tertile distribution, with the lowest tertile as the reference. Respondents´ characteristics were compared across energy-adjusted tertiles of DII using the chi-square test or analysis of variance (ANOVA) for categorical or continuous variables, respectively. Also, the characteristics of respondents were stratified by non-cases and cases of IDA using the chi-square test or independent sample t-test for categorical or continuous variables, respectively. Pearson correlation coefficients and partial correlation analyses were used to evaluate the relationships between energy-adjusted DII and Hb concentration in all respondents and by IDA status. Logistic regression was used to estimate multivariable-adjusted odds ratios (OR) and 95% confidence interval (CI) of IDA by tertiles distribution of energy-adjusted DII: second (T2) and third (T3) tertiles compared with the first/lowest tertile (T1). Model 1 was adjusted for age (years, continuous) only. Model 2 was model 1 additionally adjusted for primigravidae status (no, yes). Model 3 was model 2 additionally adjusted for monthly income (≤ N16,000, > N16,000). Test for trend was carried out by assigning the median value of DII for each tertile as a continuous variable in the models. All statistical analyses were carried out at a two-sided P<0.05 using IBM SPSS Statistics for Windows, version 23 (IBM Corporation, Armonk, NY, USA).

## Results

**Characteristics of respondents:** characteristics of respondents in this study are presented in Table 1. The mean age of the 432 respondents included in this study was 28.5 ± 4.6 years, and most respondents were ≥ 25 years of age 346 (80.9%). The mean gestational age of respondents was 31.3 ± 4.1 weeks, and 206 (47.7%) of respondents were primigravida and mean energy intake was 2822.5 ± 129.3 kcal/day and mean DII was 20.3 ± 3.3 mg/day.

**Table 1:** comparison of respondents characteristics by tertiles of energy-adjusted dietary iron intakes

			Tertiles of energy-adjusted dietary iron intakes			
Characteristic		All	T1	T2	T3	P
n			144	145	143	
Age (in years)		28.5 ± 4.6	28.2 ± 4.4	29.0 ± 4.3	28.5 ± 5.0	0.363
16 - 24		86 (19.9)	33 (38.4)	22 (25.6)	31 (36.0)	0.208
≥ 25		346 (80.1)	111 (32.1)	123 (35.5)	112 (32.4)	
Gestational age (weeks)		31.3 ± 4.1	31.4 ± 4.0	31.1 ± 4.2	31.4 ± 4.0	0.751
Primigravidae	No	226 (52.3)	78 (34.5)	77 (34.1)	71 (31.4)	0.726
Yes	Yes	206 (47.7)	66 (32.0)	68 (33.0)	72 (35.0)	
Parity		1.8 ± 0.9	1.7 ± 0.9	1.7 ± 0.9	1.9 ± 0.9	0.542
	≤2	182 (80.5)	63 (34.6)	63 (34.6)	56 (30.8)	0.597
	>2	44 (19.5)	13 (29.5)	14 (31.8)	17 (38.9)	
Education	Secondary	203 (47.3)	62 (30.5)	66 (32.5)	75 (36.9)	0.263
	Tertiary	226 (52.7)	80 (35.4)	79 (35.0)	67 (29.6)	
Marital status	Never married	20 (4.6)	04 (20.0)	09 (45.0)	07 (35.0)	0.369
	Married	411 (95.4)	140 (34.1)	135 (32.8)	136 (33.1)	
Monthly income	≤ N16,000	27 (29.2)	06 (21.4)	08 (32.1)	13 (46.4)	0.261
	> N16,000	68 (70.8)	22 (32.4)	26 (38.2)	20 (29.4)	
Dietary Fe intake (mg/day)		20.3 ± 3.3	16.6 ± 1.4	19.7 ± 1.0*	23.7 ± 2.0*	0.000
Energy (Kcal)		2822.5 ± 129.3	2841.4 ± 112.2	2789.8 ± 115.5*	2836.5 ± 151.4*	0.001
PCV (%)		31.8 ± 2.8	32.1 ± 2.6	31.7 ± 3.2	31.5 ± 2.6	0.279
Haemglobin (g/L)		97.9 ± 12.9	89.6 ± 9.1	98.4 ± 10.8	105.8 ± 13.0*	0.000
Anaemia	No	70 (16.2)	04 (2.8)	09 (6.2)	57 (39.9)	0.000
	Yes	362 (83.8)	140 (97.2)	136 (93.8)	86 (60.1)	

Continuous data are presented as mean ± SD and compared using one-way ANOVA with a least significant difference as a posthoc test (using the first tertile as reference); categorical data are presented as n (%) and compared using the chi-square test; *mean values were significantly different compared to the first tertile

**Factors associated with energy-adjusted Iron intakes of respondents:** characteristics of respondents by tertiles of energy-adjusted DII (Table 1) revealed age, gestational age, gravidae status, parity, education, marital status, income and PCV of respondents differed insignificantly by tertiles of energy-adjusted DII. However, Hb concentration was higher (P<0.001) among respondents in the third tertile of energy-adjusted DII (105.8 ± 13.0 g/L) compared to their counterparts in the first tertile (89.6 ± 9.1 g/L).

**Prevalence of IDA:** out of the 362 (83.8%) with IDA in the entire sample (Table 1), 197 (45.6%) had moderate IDA, 165 (38.2%) had mild IDA, but there was no case of severe IDA among pregnant women in this study. The proportion of subjects with IDA was significantly higher (P<0.001) among respondents in the first tertile 140 (97.2%) of energy-adjusted DII compared to respondents in the third tertile 86 (60.1%) (Table 1).

**Factors associated with IDA:** stratifying the characteristics of respondents by IDA status (Table 2), we found that the age (P=0.372) and gestational weeks (P=0.306) of the respondents were not associated with IDA status. Prevalence of IDA was insignificantly (P=0.527) higher among respondents within 16 - 24 years; 74 (86.0%) compared to those ≥ 25 years, 288 (83.2%). Primigravidae women presented a significantly (P=0.028) higher proportion of IDA 181 (87.9%) compared to multi-primigravidae women 181 (80.1%). IDA prevalence differed insignificantly with the parity, education, and marital status of respondents. Mean DII was significantly lower (P<0.001) among those with IDA (19.2 ± 2.7 mg/day) compared to respondents without IDA (23.9 ± 3.5 mg/day). Mean energy intakes were significantly higher (P=0.039) among those with IDA (2814.6 ± 113.2 kcal) compared to respondents without IDA (2863.4 ± 188.2 kcal). Furthermore, mean PCV differed insignificantly by IDA status (P=0.103), but mean Hb concentration was significantly higher (P<0.001) among respondents without IDA (116.7 ± 5.5 g/L) compared to those with IDA (94.3 ± 10.6 g/L).

**Table 2:** comparison of respondents characteristics by IDA status

		IDA status		
Characteristic		No	Yes	P-value
Age (in years)		29.0 ± 4.9	28.5 ± 4.5	0.372
	16 - 24	12 (14.0)	74 (86.0)	0.527
	≥ 25	58 (16.8)	288 (83.2)	
Gestational age (weeks)		31.8 ± 4.4	31.2 ± 4.0	0.306
Primigravidae	No	45 (19.9)	181 (80.1)	0.028
	Yes	25 (12.1)	181 (87.9)	
Parity		1.8 ± 0.9	1.7 ± 0.9	0.506
	≤*2	37 (20.3)	145 (79.7)	0.985
	>2	09 (20.5)	35 (79.5)	
Education	≤ Secondary	38 (18.7)	165 (81.3)	0.202
	Tertiary	32 (14.2)	194 (85.8)	
Marital status*	Never married	02 (10.0)	18 (90.0)	0.453
	Married	67 (16.3)	344 (83.7)	
Monthly income*	≤ N16,000	11 (42.9)	16 (57.1)	0.026
	> N16,000	14 (20.6)	54 (79.4)	
Dietary Fe intake (mg/day)		23.9 ± 3.5	19.2 ± 2.7	0.000
Energy (Kcal)		2863.4 ± 188.2	2814.6 ± 113.2	0.039
PCV (%)		31.3 ± 2.4	31.8 ± 2.9	0.103
Haemoglobin (g/L)		116.7 ± 5.5	94.3 ± 10.6	0.000

Continuous data are presented as mean ± SD and compared using the t-test; categorical data are presented as n (%) and compared using the chi-square test

**Energy-adjusted iron intakes and Hb concentration:** Pearson correlation coefficient (Table 3) of the relationship between energy-adjusted DII and Hb concentration was r = 0.588, P<0.001. The relationship (though slightly attenuated) remained after adjusting for primigravida status and monthly income (r = 0.399, P<0.0001).

**Table 3:** correlations and partial correlations between iron intakes and blood haemoglobin

	All respondents				Non-IDA				IDA			
	r^1^	P-value	r^2^	P-value	r^1^	P-value	r^2^	P-value	r^1^	P-value	r^2^	P-value
Blood haemoglobin (g/L)	0.588	0.000	0.502	0.000	0.399	0.000	0.336	0.005	0.621	0.000	0.594	0.002

r^1^: Pearson correlation coefficient; r^2^: partial correlation coefficient adjusting for primigravida and monthly income

**Energy-adjusted Iron intakes and odds of IDA:** in the logistic regression models (Table 4), unadjusted odds of IDA for the second and third tertile of energy-adjusted DII (using the first tertile as reference) were: 0.43 (0.13, 1.43) and 0.04 (0.01, 0.12) P for trend <0.0001, respectively. The odds remained after adjusting for age and gravidae status. In the final model, OR and 95% CI for odds of IDA across tertiles of energy-adjusted DII was 1.00 for the first tertile, 0.32 (0.05, 1.77) for the second tertile and 0.07 (0.01, 0.36) for the third tertile P for trend <0.0001 after adjusting for age, primigravidae status and monthly income.

**Table 4:** multivariable-adjusted odds ratio and 95% confidence interval of IDA risk by tertiles of energy-adjusted iron intakes

	Tertiles of energy-adjusted dietary iron intakes			
	T1	T2	T3	P for trend
Non-cases	04 (2.8)	09 (6.2)	57 (39.9)	
Cases	140 (97.2)	136 (93.8)	86 (60.1)	
Crude odds*	1.00 (reference)	0.43 (0.13, 1.43)	0.04 (0.01, 0.12)	<0.0001
Model 1	1.00 (reference)	0.44 (0.13, 1.47)	0.04 (0.01, 0.12)	<0.0001
Model 2	1.00 (reference)	0.42 (0.12, 1.41)	0.03 (0.01, 0.11)	<0.0001
Model 3	1.00 (reference)	0.32 (0.05, 1.77)	0.07 (0.01, 0.36)	<0.0001


* unadjusted odds; model 1: adjusted for age (in years continuous) only; model 2: model 1 additionally adjusted for primigravida status (no, yes); model 3: model 2 additionally adjusted for monthly income (≤ N16,000, > N16,000)

## Discussion

To the best of our understanding, our study presents evidence on the relationship between DII and odds of IDA among pregnant women. First, DII among pregnant women in this population was generally low, and the prevalence of IDA was high. Second, higher DII was inversely associated with odds of IDA. Our findings provided reliable data for public health advisory in managing IDA in this population. Iron deficiency anaemia (IDA) has been identified as the most common micronutrient deficiency affecting all age groups [2] and plays a pernicious role in increased morbidity and mortality among pregnant women [4].

Higher DII was inversely associated with odds of IDA in this sample. In tandem with our findings, similar reports among women of reproductive age in India [24] and school children in Tanzanian [25] have demonstrated that higher DII was associated with lower odds of IDA. Our findings, alongside previous reports [24-28], affirm the importance of adequate DII in mitigating IDA risk. In light of our findings, improving DII among women in the entire life course (particularly before pregnancy to adequately prepare the maternal iron stores for pregnancy and lactation roles) is necessary to avert IDA risk. Also, we found that pregnant women had an average daily DII of about 20.3 mg/day. This was below the 27 mg/day recommended dietary allowance for healthy pregnant women by the Food and Nutrition Board, Institute of Medicine and the National Academies of the United States [29]. This recommendation is necessary to guide appropriate public intervention strategies in the management of IDA. Despite this, DII in our study appears higher than similar estimates among a population of pregnant women in Ghana (10.94 mg/day) [30], South Africa (12.2 mg/day) [31], Iran (13.38 mg/day) [32], Europe (8.3-15.4 mg/day) [33], Jordan (14.9 mg/day) [34] and China (25.0 mg/day) [35]. The importance of adequate DII in pregnancy cannot be underestimated in maternal and child health. Some reports have linked poor DII to odds of pregnancy complications [4,5] and congenital heart defects [35].

The prevalence of IDA was high in our sample. This underscores the severity of IDA as a significant public health problem among pregnant women. Our findings differed from similar local studies [12,36-39] conducted in different regions of Nigeria, where the prevalence of IDA in pregnancy was reported. Most of these studies were conducted in tertiary and secondary health facilities (as against primary health centres in our report). Generally, health-seeking patronage among pregnant women in this setting is low. This is further complicated by poor health service delivery because of a lack of expertise, functional equipment and basic inventory [40]. Iron deficiency anaemia (IDA) in pregnancy may likely have been under-reported over the years, given that most pregnant women would prefer seeking health advice outside the secondary and tertiary health facilities [41]. In tandem with this observation, a recent study in Abeokuta, Nigeria [37] has reported a high prevalence of IDA among pregnant women patronizing traditional birth homes. Aside from variation in DII, the quality of care and health advisory [41,42] undoubtedly aggravated the burden of IDA in this sample. Howbeit a country-wide study is necessary to gain further insights into the burden of IDA in pregnancy in Nigeria.

Similarly, IDA prevalence in our study was higher than that observed in a similar population in Ethiopia [10,43-46], Tanzania [11], Singapore [47], Iran [48] and Uganda [49]. Also, most IDA cases in our report were moderate. This finding is similar to reports from Kenya [50] and Southern Ethiopia [51]. These differences can be explained in several ways. First, most of these reports assessed IDA in early pregnancy when the risk is likely lower. In our report, we assessed IDA ≈ 24 weeks into pregnancy. Second, IDA risk is a function of socioeconomic and environmental factors that vary across populations. Third, DII and iron supplementation differ by population. However, our findings underscore the importance of health promotion programmes to improve dietary intakes of essential nutrients and micronutrient supplementation pre-pregnancy, during pregnancy and post-pregnancy to improve maternal iron stores.

Furthermore, the prevalence of IDA was higher among primigravidae than multigravidae in our study. This observation was in tandem with some studies [12,48] but differed from others [51,52]. On one hand, the number of pregnancies is unlikely to promote IDA risk in pregnancy, but rather the quantum of preparation among mothers is a vital factor in determining IDA risk in pregnancy. In tandem with the observation, Xing *et al*. [53] observed that lack of preparation for pregnancy was associated with IDA in the first pregnancy. On the other hand, prior pregnancy experience and health education among multigravidae women explain the significantly low IDA prevalence. This implies that the likelihood of IDA risk in pregnancy is related to the extent of pre-pregnancy preparation. This is a critical window of opportunity to engage public intervention in managing IDA.

In our study, monthly income was associated with IDA risk in pregnancy. Pregnant women with higher income are likely to present a higher proportion of IDA. This result is inconsistent with previous studies [49,51,54], which found IDA rampant among women with low income due to a lack of resources to afford good health services. However, IDA is a function of nutritional deficiency than health services. Taken together, interventions promoting the consumption of iron-rich foods might be a viable, cost-effective primary prevention strategy to reduce the burden of IDA among women.

**Limitations:** our study has both limitations and strengths. The cross-sectional design exempts the inference of a causal relationship between DII and IDA. Also, the generalizability of our findings is limited given we conducted the study in the Ifako-Ijaiye area of Lagos State only. DII was reported using 24-hour dietary recall at a single time point and may not represent overall diet exposure. Residual confounding or unmeasured factors are to be considered in evaluating our results. Future cohort studies exploring the implication of DII and IDA risk are necessary.

## Conclusion

Our data suggest that higher dietary iron intake was associated with lower odds of IDA among pregnant women in this population.

### What is known about this topic

- Iron deficiency anaemia (IDA) is a prominent risk factor for poor pregnancy outcomes and maternal mortality;
- Data on the burden of IDA in the general population has been reported, but little is known about the level of dietary iron intake among pregnant women.

### What this study adds

- Dietary iron intakes among pregnant women in this sample were low;
- Higher dietary iron intake was inversely associated with the odds of IDA among pregnant women in this sample;
- Promoting iron-rich foods in pregnancy and the entire life course might be a viable dietary strategy to alleviate IDA risk among women.

## References

[1] World Health Organization (2017). Nutritional anaemias: tools for effective prevention and control.

[2] Lopez A, Cacoub P, Macdougall IC, Peyrin-Biroulet L (2016). Iron deficiency anaemia. Lancet.

[3] Khaskheli M-N, Baloch S, Sheeba A, Baloch S, Khaskheli FK (2016). Iron deficiency anaemia is still a major killer of pregnant women. Pak J Med Sci.

[4] Daru J, Zamora J, Fernández-Félix BM, Vogel J, Oladapo OT, Morisaki N (2018). Risk of maternal mortality in women with severe anaemia during pregnancy and post partum: a multilevel analysis. Lancet Glob Health.

[5] Brabin BJ, Hakimi M, Pelletier D (2001). An Analysis of anemia and pregnancy-related maternal mortality. J Nutr.

[6] Rebecca J, Stoltzfus LM, Black RE, Rebecca J, Stoltzfus LM, Black RE (2004). Chapter 3: iron deficiency anaemia. Comparative quantification of health risk, global and regional burden of diseases attributable to selected major risk factors.

[7] National Population Commission (NPC) (Nigeria), Inner City Fund International (ICF) (2019). Nigeria demographic and health survey 2018.

[8] Wieringa FT, Sophonneary P, Whitney S, Mao B, Berger J, Conkle J (2016). Low Prevalence of iron and vitamin A deficiency among Cambodian women of reproductive age. Nutrients.

[9] Erhabor O, Muhammad AD, Adias TC, Ahmed Y, Erhabor T (2020). Anaemia and thrombocytopenia among pregnant women attending Aminu Kano Teaching Hospital, Kano State, North Western Nigeria. Hum Antibodies.

[10] Lebso M, Anato A, Loha E (2017). Prevalence of anemia and associated factors among pregnant women in Southern Ethiopia: a community based cross-sectional study. PLoS One.

[11] Stephen G, Mgongo M, Hussein Hashim T, Katanga J, Stray-Pedersen B, Msuya SE (2018). Anaemia in pregnancy: prevalence, risk factors, and adverse perinatal outcomes in Northern Tanzania. Anemia.

[12] Olubukola A, Odunayo A, Adesina O (2011). Anemia in pregnancy at two levels of health care in Ibadan, south west Nigeria. Ann Afr Med.

[13] (2012). United Nations Human Settlements Programme (UN-HABITAT).

[14] Charan J, Biswas T (2013). How to calculate sample size for different study designs in medical research. Indian J Psychol Med.

[15] McLean E, Cogswell M, Egli I, Wojdyla D, de Benoist B (2009). Worldwide prevalence of anaemia, WHO vitamin and mineral nutrition information system, 1993-2005. Public Health Nutr.

[16] De Benoist B, Cogswell M, Egli I, McLean E (2008). Worldwide prevalence of anaemia 1993-2005: WHO global database on anaemia.

[17] Kothari CR (2004). Research methodology: methods and techniques.

[18] Beer-Borst S, Amadò R (1995). Validation of a self-administered 24-hour recall questionnaire used in a large-scale dietary survey. Z Ernahrungswiss.

[19] Cambridge Biomedical Research Centre DAPA measurement toolkit: 24-hour dietary recalls.

[20] Food and Agriculture Organization of the United Nations (2004). Uses of food consumption and anthropometric surveys in the Caribbean: how to transform data into decision-making tools.

[21] Stadlmayr B, Charrondiere UR, Enujiugha VN, Bayili RG, Fagbohoun EG, Samb B (2012). West African food composition table/table de composition des aliments d´Afrique de l´Ouest.

[22] Willett W, Stampfer MJ (1986). Total energy intake: implications for epidemiologic analyses. Am J Epidemiol.

[23] Cheesbrough M (2006). District laboratory practice in tropical countries, part 2.

[24] Swaminathan S, Ghosh S, Varghese JS, Sachdev HS, Kurpad AV, Thomas T (2019). Dietary iron intake and anemia are weakly associated, limiting effective iron fortification strategies in India. J Nutr.

[25] Tatala S, Ndossi G, Svanberg U, Ash D (2004). Impact of dietary iron intake on anaemia in Tanzanian schoolchildren. South Afr J Clin Nutr.

[26] Prentice AM, Mendoza YA, Pereira D, Cerami C, Wegmuller R, Constable A (2016). Dietary strategies for improving iron status: balancing safety and efficacy. Nutr Rev.

[27] Bailey RL, Pac SG, Fulgoni VL III, Reidy KC, Catalano PM (2019). Estimation of total usual dietary intakes of pregnant women in the United States. JAMA Netw Open.

[28] Jarrah SS, Halabi JO, Bond AE, Abegglen J (2007). Iron deficiency anemia (IDA) perceptions and dietary iron intake among young women and pregnant women in Jordan. J Transcult Nurs.

[29] Meyers LD, Hellwig JP, Otten JJ (2006). Dietary reference intakes: the essential guide to nutrient requirements.

[30] Ayensu J, Annan R, Lutterodt H, Edusei A, Peng LS (2020). Prevalence of anaemia and low intake of dietary nutrients in pregnant women living in rural and urban areas in the Ashanti region of Ghana. PLoS One.

[31] Motadi SA, Matsea Z, Mogane PH, Masidwali P, Makwarela M, Mushaphi L (2020). Assessment of nutritional status and dietary intake of pregnant women in rural area of Vhembe District, Limpopo Province. Ecol Food Nutr.

[32] Hajianfar H, Abbasi K, Azadbakht L, Esmaeilzadeh A, Mollaghasemi N, Arab A (2020). The association between maternal dietary iron intake during the first trimester of pregnancy with pregnancy outcomes and pregnancy-related complications. Clin Nutr Res.

[33] Milman NT (2020). Dietary iron intake in pregnant women in Europe: a review of 24 studies from 14 countries in the period 1991-2014. J Nutr Metab.

[34] Tayyem RF, Allehdan SS, Alatrash RM, Asali FF, Bawadi HA (2019). Adequacy of nutrients intake among Jordanian pregnant women in comparison to dietary reference intakes. Int J Environ Res Public Health.

[35] Yang J, Kang Y, Cheng Y, Zeng L, Shen Y, Shi G (2020). Iron intake and iron status during pregnancy and risk of congenital heart defects: a case-control study. Int J Cardiol.

[36] Adanikin AI, Awoleke JO (2016). Sociodemographic factors associated with anaemia in pregnancy at booking for antenatal care. J Obstet Gynaecol.

[37] Idowu OA, Mafiana CF, Dapo S (2005). Anaemia in pregnancy: a survey of pregnant women in Abeokuta, Nigeria. Afr Health Sci.

[38] Okoh DA, Iyalla C, Omunakwe H, Iwo-Amah RS, Nwabuko C (2016). A retrospective study of the prevalence of anaemia in pregnancy at booking in Niger Delta, Nigeria. J Obstet Gynaecol.

[39] Ugwu EO, Dim CC, Uzochukwu BS, Iloghalu EI, Ugwu AO (2014). Malaria and anaemia in pregnancy: a cross-sectional study of pregnant women in rural communities of Southeastern Nigeria. Int Health.

[40] Gage AJ, Ilombu O, Akinyemi AI (2016). Service readiness, health facility management practices, and delivery care utilization in five states of Nigeria: a cross-sectional analysis. BMC Pregnancy Childbirth.

[41] Onyeneho NG, Igweonu OU (2016). Anaemia is typical of pregnancies: capturing community perception and management of anaemia in pregnancy in Anambra State, Nigeria. J Health Popul Nutr.

[42] Oyekale AS (2017). Assessment of primary health care facilities' service readiness in Nigeria. BMC Health Serv Res.

[43] Berhe B, Mardu F, Legese H, Gebrewahd A, Gebremariam G, Tesfay K (2019). Prevalence of anemia and associated factors among pregnant women in Adigrat General Hospital, Tigrai, Northern Ethiopia, 2018. BMC Res Notes.

[44] Grum T, Brhane E, Hintsa S, Kahsay G (2018). Magnitude and factors associated with anemia among pregnant women attending antenatal care in public health centers in central zone of Tigray region, northern Ethiopia: a cross sectional study. BMC Pregnancy Childbirth.

[45] Gudeta TA, Regassa TM, Belay AS (2019). Magnitude and factors associated with anemia among pregnant women attending antenatal care in Bench Maji, Keffa and Sheka zones of public hospitals, Southwest, Ethiopia, 2018: a cross-sectional study. PLoS One.

[46] Kebede A, Gerensea H, Amare F, Tesfay Y, Teklay G (2018). The magnitude of anemia and associated factors among pregnant women attending public institutions of Shire Town, Shire, Tigray, Northern Ethiopia, 2018. BMC Res Notes.

[47] Loy SL, Lim LM, Chan SY, Tan PT, Chee YL, Quah PL (2019). Iron status and risk factors of iron deficiency among pregnant women in Singapore: a cross-sectional study. BMC Public Health.

[48] Mardani M, Rezapour S, Ahmadipour S, Mohsenzadeh A, Khalkhali Rad A, Roosta S (2017). Prevalence of anemia and its risk factors among pregnant women in Khorramabad (Iran) 2010-2014. J Matern Fetal Neonatal Med.

[49] Obai G, Odongo P, Wanyama R (2016). Prevalence of anaemia and associated risk factors among pregnant women attending antenatal care in Gulu and Hoima Regional Hospitals in Uganda: a cross sectional study. BMC Pregnancy Childbirth.

[50] Okube OT, Mirie W, Odhiambo E, Sabina W, Habtu M (2016). Prevalence and factors associated with anaemia among pregnant women attending antenatal clinic in the second and third trimesters at Pumwani Maternity Hospital, Kenya. Open Journal of Obstetrics and Gynecology.

[51] Gedefaw L, Ayele A, Asres Y, Mossie A (2015). Anaemia and associated factors among pregnant women attending antenatal care clinic in Walayita Sodo town, Southern Ethiopia. Ethiop J Health Sci.

[52] Ivoke N, Eyo JE, Ivoke ON, Nwani CD, Odii EC, Asogwa CN (2013). Anaemia prevalence and associated factors among women attending antenatal clinics in South-Western Ebonyi State, Nigeria. International Journal of Medicine and Medical Sciences.

[53] Xing Y, Yan H, Dang S, Zhuoma B, Zhou X, Wang D (2009). Hemoglobin levels and anemia evaluation during pregnancy in the highlands of Tibet: a hospital-based study. BMC Public Health.

[54] Siteti MC, Namasaka SD, Ariya OP, Injete SD, Wanyonyi WA (2014). Anaemia in pregnancy: prevalence and possible risk factors in Kakamega County, Kenya. Science Journal of Public Health.

